# A Graph is Worth a Thousand Words: How Overconfidence and Graphical Disclosure of Numerical Information Influence Financial Analysts Accuracy on Decision Making

**DOI:** 10.1371/journal.pone.0160443

**Published:** 2016-08-10

**Authors:** Ricardo Lopes Cardoso, Rodrigo Oliveira Leite, André Carlos Busanelli de Aquino

**Affiliations:** 1 Fundação Getulio Vargas, Brazilian School of Public and Business Administration, Rio de Janeiro, RJ, Brazil; 2 Universidade de São Paulo, School of Economics, Business Administration and Accounting at Ribeirão Preto, Ribeirão Preto, SP, Brazil; Universitat Jaume I, SPAIN

## Abstract

Previous researches support that graphs are relevant decision aids to tasks related to the interpretation of numerical information. Moreover, literature shows that different types of graphical information can help or harm the accuracy on decision making of accountants and financial analysts. We conducted a 4×2 mixed-design experiment to examine the effects of numerical information disclosure on financial analysts’ accuracy, and investigated the role of overconfidence in decision making. Results show that compared to text, column graph enhanced accuracy on decision making, followed by line graphs. No difference was found between table and textual disclosure. Overconfidence harmed accuracy, and both genders behaved overconfidently. Additionally, the type of disclosure (text, table, line graph and column graph) did not affect the overconfidence of individuals, providing evidence that overconfidence is a personal trait. This study makes three contributions. First, it provides evidence from a larger sample size (295) of financial analysts instead of a smaller sample size of students that graphs are relevant decision aids to tasks related to the interpretation of numerical information. Second, it uses the text as a baseline comparison to test how different ways of information disclosure (line and column graphs, and tables) can enhance understandability of information. Third, it brings an internal factor to this process: overconfidence, a personal trait that harms the decision-making process of individuals. At the end of this paper several research paths are highlighted to further study the effect of internal factors (personal traits) on financial analysts’ accuracy on decision making regarding numerical information presented in a graphical form. In addition, we offer suggestions concerning some practical implications for professional accountants, auditors, financial analysts and standard setters.

## Introduction

The study of interpretation of graphical information by accountants is not something new. Literature suggests that graphs can aid in the communication of accounting information [1]. Indeed graphs are shown to improve understanding of information and accuracy of forecast judgments [2]. Multidimensional graphs are also shown to improve judgment and decision-making processes [3]. However, not every way of presenting information has the same effect on users of accounting information. Kloptchenko et al. [4] notices that it is difficult to extract meaning from textual disclosure. Vessey [5] argued that spatial information is better represented with graphs, while symbolic information is better represented with tables. Peterson [6] investigated the relationship of readers’ retention, reaction and reading time with four different presentation methods (narrative only, narrative with the aid of tables, narrative with the aid of graphs, and narrative with the aid of tables and graphs). Based on a sample of 625 students, Peterson identified that narrative with the aid of tables helps readers’ retention most, followed by narrative with the aid of graphs, then narrative with the aid of tables and graphs. Male students preferred narrative with the aid of graphs, and female students preferred narrative with the aid of tables. Concerning reading time, students took the least amount of time to read the report if they were reading narrative with the aid of tables; the method that took the longest to read was narrative only. Ten years after Peterson’s research, Kelly [7] investigated the effects of display format (text, tables and column graphs) and data density on time spent reading statistics. Using a sample of 18 journalism undergraduate students, Kelly identified that data format did not affect accuracy. However, display format caused significant effect on time spent; i.e., both tables and graphs were less time-consuming than text. A recent study [8] showed that interactivity, visualization and difficulty affects the decision-making process of accountants and financial analysts. However, there is a gap in the literature: no study has analyzed both intrinsic characteristics of the individual and how it affects interpretation of graphic information.

In this study, we examine whether four different types of information disclosure (text, table, line graph and column graph) can help or harm financial analysts’ accuracy on the interpretation of numerical information, and we investigate the role of overconfidence in such decision-making processes.

Our study attempts to provide more relevant evidence for accounting and finance settings than previous studies, once we used a sample comprised by financial analysts instead of students. Secondly, no previous study used textual disclosure (narrative only) as basement measure to provide a comparison between the different types of graphical information disclosure (graph only). Third, we also investigate whether intrinsic characteristics of the individual alter the interpretation of different ways of disclosing information. Previous research [9] has provided evidence that intrinsic characteristics of an accountant can alter her/his decision-making process. This study will focus on overconfidence, a trait that can be divided into three subtypes: overestimation, overplacement and overprecision [10, 11]. In this study we use overplacement, which can be described as “the overplacement of one’s performance relative to others” [12]. The “overconfidence trap” can lead to “errors in judgment and, in turn, bad decisions” [13].

Thus, our study aims to assess whether different disclosure practices of numerical information (text, table, column graph and line graph) affect accuracy of financial analysts. Additionally, we investigate whether personal characteristics (overconfidence and gender) also affect their accuracy. Based on a 4×2 mixed-design experiment, this study provides empirical evidence from 295 professional accountants who work as financial analysts that different ways of disclosing the same information can increase (or decrease) accuracy of judgment and decision making. Specifically, information disclosed on column graphs are significantly more correctly interpreted by financial analysts than textual disclosure, followed by line graphs. There was no difference in accuracy when respondents analyzed tables or text. Regarding overplacement, we identified that overconfident financial analysts presented more wrong answers (and less correct answers) than non-overconfident financial analysts, regardless of the type of information disclosure and of respondent’s gender.

The study of graph interpretation ability by accountants and financial analysts is relevant in the accounting context because most modern annual reports contain graphs. For example, the International Accounting Standards Board (IASB) recently amended the International Financial Reporting Standard 7 (IFRS 7)–*Financial Instruments*: *Disclosures* requiring that “if the quantitative data disclosed as at the end of the reporting period are unrepresentative of an entity’s exposure to risk during the period, an entity shall provide further information that is representative” (IFRS 7, par. 35). If this is the case, then implementation guidance exhorts the presentation of graphs: “[…] if an entity typically has a large exposure to a particular currency, but at year-end unwinds the position, the entity might disclose a graph that shows the exposure at various times during the period […]” (IFRS 7, par. IG20).

In fact, because standard-setters urge entities to present graphs in the notes, the use of graphs in financial reporting might increase significantly. Therefore, knowledge of individuals’ ability to interpret graphs and the impact of their personal characteristics in performing such a task becomes very important for preparers and auditors of financial reports, financial analysts, standard-setters and accounting professors, and it may enhance impression management literature in many venues.

Additionally, impression management literature suggests that graphs are more vulnerable to manipulation because they are non-audited and not prescribed [1, 14, 15]. Three main types of impression management through graphs were investigated in previous researches. These are: selectivity (i.e., occurs when a company deliberately chooses graphs so that they will convey a favorable impression of the company); measurement distortion (i.e., the figures on the graphs do not accurately represent the underlying financial data); and presentational enhancement (i.e., graphs are constructed so as to emphasize certain design features) [1, 14–18]. For example, Beattie and Jones [1] analyzed annual reports from 1989 of 240 U.K. listed companies and identified that 79% of them used graphs, and the majority were column graphs (64%). In addition to the usage of graphs, they identified that the presence (absence) of graph is associated with a positive (negative) trend on corporation’s performance. Regarding distortion, they identified that in 73% of the cases material discrepancies were identified, i.e., graphical trends were exaggerated rather than understated. Later, Beattie and Jones [14] extended such analysis for a sample of 300 domestically-listed only enterprises in France, Germany, Australia, the Netherlands, the U.K., and the U.S., over a 5-years period ending on 1992. They identified that the graphs are often used to enhance the perception of good news.

Knowledge of individuals’ ability to interpret information represented in many different forms (e.g., text, table, line graph, and column graph) may enhance impression management literature in many venues. For example, the selectivity type of impression management could be investigated in depth and far beyond the traditional approach [15, 19]. For instance, selectivity could also be investigated as the choice of the graph format (e.g., line, column or pie chart), or presenting a table or only text (narrative) instead of a graph.

The remaining sections of this paper are organized as follows; the next section presents literature review and hypotheses development. Section 3 describes the experiment design, data collection process and methods to test hypotheses. Section 4 presents and discusses the results for each hypothesis. Finally, section 5 presents final remarks and suggestions for further research.

## Literature Review and Hypotheses Development

Different types of accounting information disclosure by companies are shown to change the decision-making process of both financial analysts [20] and investors [21]. It also affects the confidence interval of forecasts [22]. In an experiment with undergraduate students, Beattie and Jones [23] showed that students perceived a company whose graphs had received a measurement distortion as better than the same company if such graphs had not been distorted. The same conclusion was achieved by Arunachalam, Pei and Steinbart [18], with a total sample of 126 students across three experiments. In addition, companies that face poor performance use different disclosure techniques in order to manipulate the market’s perception of their performance [24].

Different ways of presenting financial information are shown to influence the accuracy of decision making on different accounting tasks [8, 25]. Both researches use experimental empirical evidence to reach the same conclusion as Vessey’s [5]: different types of information disclosure have different effects in understanding performance in different situations. Only two papers compare textual with graphical disclosure: Peterson [6] and Kelly [7], but under different methodological approaches. Our research differs from Peterson’s in the following perspectives: (i) we compared the effect of four different presentation formats (text/narrative only, table only, column graph only and line graph only) individually. On the other hand, Peterson compared text/narrative only with text/narrative with the aid of tables, or graphs, or tables and graphs. (ii) We analyzed the effects of two different graph formats (column and line), but Peterson did not explicit which format of graph he presented to subjects. (iii) We measured the effect in terms of correct answers (accuracy as dependent variable), Peterson did not measure accuracy, instead she measured three dependent variables: retention (memory), reaction (preference concerning visual appeal) and time spent on performing the task. On the other hand, our research differs from Kelly’s in the following perspectives: (i) we did not investigate time consumption or another measure of efficiency, but only accuracy (a measure of effectiveness)–we did so because we collected data through a web-based questionnaire (not in a laboratory environment), hence we could not presume that respondents were not performing alternative tasks at the same time and that concurrent visual stimuli were minimal. (ii) The only graphical format manipulated by Kelly was column graph, and we manipulated two formats, column graph and line graph.

Based on results provided by previous literature [6, 7, 23] we propose that different forms of information disclosure (text, table, column graph and line graph) differently affect informational perception and accuracy on decision making of financial analysts. Hence, numerical information disclosed on tables or graphs might enhance accuracy, as follows:

**H1A:** *Numerical information disclosed on tables enhances accuracy when compared to text (narrative) only disclosure*.

**H1B:** *Numerical information disclosed on graphs enhances accuracy when compared to text (narrative) only disclosure*.

We do not propose any hypothesis on comparisons between tables and graphs, nor between column graphs and line graphs, because of the lack of previous evidence or any reasonable argument about this in previous literature.

There is empirical evidence that overconfident individuals will commit more errors, and that gender has a role in this intrinsic characteristic: males are more overconfident (and commit more errors) than females. Economics undergraduate male students are shown to be more overconfident than females [26] and male dealers trade with more overconfidence than females [27]. A cross-cultural study in the US, Germany, Italy and Thailand has confirmed that women financial analysts were more risk-averse than male financial analysts [28]. In addition, there is experimental evidence for differences in risk aversion and bargaining between males and females, though they are not correlated [29]. In this study we use the overplacement subdimension of overconfidence, that can be described as “the overplacement of one’s performance relative to others” [12]. As a result, we have postulated the following hypotheses:

**H2:** *Overconfident financial analysts commit more errors than non-overconfident ones*.

**H3:** *Male financial analysts are more overconfident than females*.

In order to test those hypotheses we applied an online survey experiment using Surveymonkey, as described in the next section. It can be a way to improve both internal validity and external validity [30] since it is a randomized experiment with professional financial analysts who actually use graphs to make decisions on a day-to-day basis.

## Methodological Approach

### Data collection and experiment design

Data were collected via an electronic questionnaire applied by the Brazilian Accounting Association (BAA). In Portuguese, it is called Conselho Federal de Contabilidade (CFC)– www.cfc.org.br. The research proposal of this study was submitted to and approved by the Brazilian Accounting Association Ethics Committee (*Comitê de Ética do Conselho Federal de Contabilidade*). Hence, the President of the BAA sent an electronic message containing the web link to the questionnaire to professional accountants who were already registered with it in August 2012, inviting them to take part in this research as respondents. All the volunteer respondents agreed to participate in the experiment, and data was collected anonymously. Based on the respondents’ expertise, they were required to answer a specific set of questions. Actually, those who presented themselves as financial analysts were required to answer questions related to graph interpretation. In all, 295 professional accountants whose main duties are related to financial analysis comprise the sample for this research. The sample was randomly classified among four subsamples to which 1 of 4 types of information disclosure was presented conveying the same informational content with regard to the number of people going in and out of a store during a 12 minute time period, as depicted in S1 Text, S1 Table and S1 and S2 Figs. In each case, numerical information was preceded by the following description: *The following paragraph* (text condition) */ table* (table condition) */ graph* (line graph and column graph conditions) *depicts the number of people going in and out of a store in a 12 minutes time period*.

Hence, it is a 4×2 mixed-design experiment (four types of information disclosure between subjects: textual information, table, line graph and column graph; and two questions within subjects: *In which minute is there the largest number of people entering the store?* and *In which minute is there the largest number of people exiting the store?*). The subjects were randomly assigned to one of the four types of information presentation with a probability of .40 being assigned to the line graph and a probability of .20 being assigned to one of the other three conditions. We assigned more respondents to the line graph condition because, in previous researches, line graph is less investigated than column graph and table [1, 7, 19]. Notice that we performed a robustness check with a randomly selected subsample of line graph respondents (i.e., balanced sample) and the results were robust, and significance levels and effects remained similar to the unbalanced sample, as depicted in S2 Table. Table 1 presents data descriptive statistics.

**Table 1:** Descriptive statistics and correlations.

			Correlations				
	Mean	SD	Gender	Age	Residence	Overc.	Correct Answers
**Gender**	0.6565	0.4753	1				
**Age**	39.024	10.559	.2028***	1			
**Residence**	0.6677	0.4718	.0560	-.0005	1		
**Overconfidence**	0.3559	0.4796	.0011	-.0313	-.0356	1	
**Correct Answers**	1.6102	0.7099	-.0152	-.0333	.0210	-.1594**	1
**Line Graph**	0.4203	0.4945	-.0058	-.0085	-.1185*	.0103	.0340
**Column Graph**	0.2441	0.4303	-.0211	-.0943	.0295	-.0613	.0687
**Table Graph**	0.1627	0.3697	-.0362	.0149	.0495	.0042	.0050
**Text**	0.1729	0.3788	.0666	.1038	.0731	.0522	-.1272*

Table 1 depicts data descriptive statistics and their correlations.

*p < .05

**p < .01

***p < .001. We excluded the correlations between the four randomized conditions.

We used a “neutral” problem in our experiment to avoid “expert bias” [31], which can lead experts to use System 1 (impulsivity) to understand and solve situations which they are familiarized with, while non-experts would need to use System 2 (reflectivity) because they are not familiarized with and do not face the same situations on a daily basis [32]. The “neutral” task performed in our experiment was interpreting the flow of people entering in and exiting from a store during a short period of time (12 minutes), in four different formats (text, table, line graph and column graph) as depicted in S1 Text, S1 Table and S1 and S2 Figs, respectively, and explained in the next section.

After the experiment, using the same electronic questionnaire, respondents were asked if they thought they would be in the “10% group” that responded correctly to both questions. Those who answered yes were coded as overconfident, following the literature [16–18].

### Randomization test

To test if the randomization of the four types of information presentation worked properly, three chi-squared tests were performed. In the first test, we tested distribution of types of information disclosure regarding the age of participants. In the second test it was regarding their gender. The third test was in reference to the participants’ city of residence (state capital or other). Since age is a continuous variable, it needed to be converted to a discreet variable for the test to be performed. Therefore, age was converted into a categorical variable: Junior (below 31 years), Experienced (between 31 and 47 years) and Senior (above 47 years). We control for age because there could be a bias for more senior analysts. We also control for city of residence because financial analysts living in capitals where large companies are located have more and easier access to training and other opportunities to develop their professional skills than those living in smaller cities. Gender control is important in our study because it is the independent variable analyzed in H3.

After the age variable transformation, a total of three chi-squared tests were performed and the p-values were non-significant ($\chi_{age}^{2}(6)=5.97,\;p=.427$; $\chi_{gender}^{2}(3)=1.39,\;p=.707$; $\chi_{residence}^{2}(3)=4.18,\;p=.243$), showing that there was no bias of gender, age or place of residence in the subject’s assignment in each one of the four presentation formats (between subject conditions). This suggests that the randomization worked well and provides evidence that our assignment was not biased.

Due to the fact that the analyzed conditions (i.e., types of information disclosure) were randomized in the sample, it eliminates the problem of endogeneity and self-selection, reducing systematic bias significantly. However, there may be a possible confounder in this study regarding overconfidence. Maybe some ways of disclosing numerical information can influence the participant’s overconfidence causing bias. Aiming to assess whether this was an issue in this study, another chi-squared test was performed and the result was non-significant (*χ*^2^(3) = 1.52, *p* = .678), suggesting that manipulation did not affect the participants’ overconfidence. In addition, this provides more evidence that the randomization process worked well, since there was no meaningful difference in the participants’ overconfidence. However, as depicted in Table 1, our sample female accountants tended to be older than male accountants (correlation 0.2028, p < .001).

## Results

First, the two questions asked to respondents were grouped in one variable that could assume the value of 0 (no correct answer), 1 (one correct answer) or 2 (both questions answered correctly) for the results to be estimated in one statistical test. Aiming to compare the four experimental conditions and test H1A, H1B and H2, we estimated the following ordered logit model:

$$Correct=\beta_{0}+\beta_{1}Overconf+\beta_{2}Line+\beta_{3}Column+\beta_{4}Table+\varepsilon$$

Where *Correct* denotes the number of correct answers (0, 1 or 2), *Overconf* is a dummy for overconfidence (1 = overconfident). *Line* is a dummy that assumed 1 if the respondent received a line graph, *Column* is a dummy that assumed 1 if the respondent received a column graph, and *Table* is a dummy that assumed 1 if the respondent received a table. Therefore, the baseline category is the text disclosure. We used the ordered logit model, since *Correct* is a discreet hierarchical variable. The coefficients from the ordered logit model are easier to interpret than the ordered probit, since they are the natural logarithm of the odds ratio.

Notwithstanding, we used ordered probit model and OLS in addition to ordered logit model to investigate whether table, column graph or line graph enhance accuracy at most in comparison with text (narrative only). In order to facilitate the interpretation of results, we parsimoniously added independent variables as follows. For each statistical technique (OL, OP, OLS), we first tested a univariate model (Correct = β_0_ + β_1_ Overconf + ε); i.e., models 1, 4 and 7 depicted in Table 2. Then, we added the dummy variables for disclosure type, and tested the main model (H1A, H1B and H2) (models 2, 5 and 8 depicted in Table 2). Finally, we added a dummy variable for gender to test the expanded model (Correct = β_0_ + β_1_ Overconf + β_2_ Line + β_3_ Column + β_4_ Table + β_5_ *Gender* + ε), depicted in Table 2 under the label model 3, model 6 and model 9; where *Gender* is a dummy that assumed 1 if the respondent is male. This variable is included in the model to test if males and females had different performances in the task, since our H3 hypothesizes that males are more overconfident than females and H2 hypothesizes that overconfidence harms decision making.

**Table 2:** Correct answers as a function of overconfidence, disclosure type and gender (Results from H1A, H1B and H2).

	Ordered Logit			Ordered Probit			OLS		
	Model 1	Model 2	Model 3	Model 4	Model 5	Model 6	Model 7	Model 8	Model 9
Overconfidence	-.705***	-.679**	-.681**	-.420***	-.402**	-.402**	-.237***	-.226***	-.226***
	(.269)	(.271)	(.271)	(.157)	(.158)	(.158)	(.085)	(.085)	(.085)
Line Graph		.694**	.692**		.387*	.386*		.214*	.214*
		(.345)	(.345)		(.207)	(.207)		(.117)	(.117)
Column Graph		1.046**	1.043**		.533**	.532**		.259**	.258**
		(.421)	(.421)		(.240)	(.241)		(.128)	(.129)
Table		.545	.541		.325	.323		.200	.199
		(.423)	(.414)		(.253)	(.253)		(.140)	(.141)
Gender (1 = male)			-.061			-.020			-.009
			(.285)			(.164)			(.086)
N	295	295	295	295	295	295	295	295	295
Chi² / F †	6.84***	13.67***	13.71**	7.17***	12.60**	12.62**	7.75***	3.09**	2.47**
Pseudo-R**²** / AdjR² ‡	0.016	0.031	0.031	0.016	0.029	0.029	0.026	0.028	0.024

Table 2 depicts the results from the ordered logit model, ordered probit and OLS regression. Standard Errors in parenthesis.

* p < .1

** p < .05

*** p < .01

All interactions in all models are nonsignificant.

† Chi²-test for the Ordered Logit and Ordered Probit models and F-test for the OLS models.

‡ Pseudo-R² for the Ordered Logit and Ordered Probit models and AdjR² for the OLS models.

Table 2 presents the results from the ordered logit model, as well as the ordered probit and OLS regression for comparison purpose, since the OLS coefficients are the true difference between the groups, while the ordinal logit coefficients are the natural logarithm of the odds ratio, and the ordinal probit coefficients are based on transformation of the normal curve CDF. Supporting H2, the coefficient for the overconfidence was negative and significant (*β*_1_ = −0.705, *p* < .001).

Table 3 presents the percentages of correct answers per cluster of respondents (overconfident and non-overconfident financial analysts), and the respective chi-squared test. It shows that overconfident financial analysts committed more errors than non-overconfident ones in this experiment setup. The table shows that 79% of non-overconfident respondents answered correctly both questions, while only 66% of the overconfident ones achieved such performance. Moreover, 20% of the overconfident analysts answered both questions incorrectly, while only 9% of the non-overconfident ones missed both questions.

**Table 3:** Number of correct answers by overconfidence (Results from H2).

	Number of Correct Answers			
Overconfidence	0	1	2	Total
**No**	18	22	150	190
** **	9.47%	11.58%	78.95%	100.00%
**Yes**	21	15	69	105
** **	20.00%	14.29%	65.71%	100%
**Total**	39	37	219	295
** **	13.22%	12.54%	74.24%	100.00%
Pearson *χ*^2^(2) = 7.66, p = .022				

Table 3 depicts the percentages of correct answers per cluster of respondents and the respective chi-squared test.

**Fig 1:**
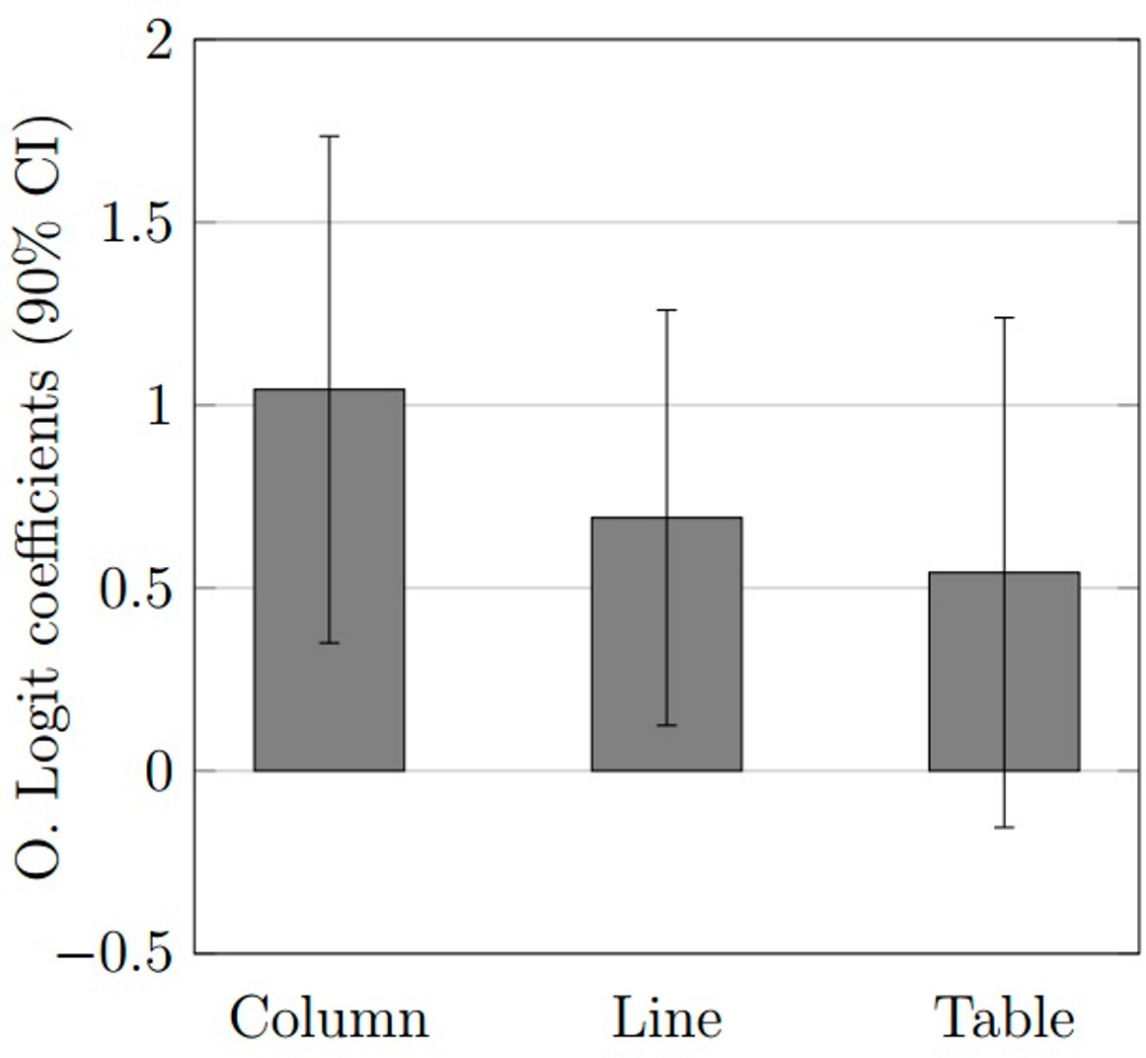
Improvement on accuracy of graphs and table versus text.

Fig 1 presents the comparative effect of different types of information disclosure (using textual–narrative only–disclosure as baseline) on financial analysts’ accuracy. Fig 1 reinforces results depicted in Table 2 that the type of information disclosure that provided the highest coefficient when compared to text was the column graph, followed by the line graph. The table coefficient did not significantly improve accuracy on decision making of financial analysts when compared to textual disclosure.

As results have shown, column graph was the best way of providing numerical flow information to individuals, followed by line graph, supporting H1B for both column graph and line graph. On the other hand, table representation did not enhance accuracy on decision making when compared to text disclosure (not supporting H1A). The result for H1A is not aligned with results presented by Peterson [6]; however, Peterson compared accuracy on interpretation of information disclosed on text (narrative only), with information disclosed on narrative with the aid of tables, narrative with the aid of graphs, and narrative with the aid of tables and graphs. Such methodological idiosyncrasy may lead to different results because the *text plus table* approach adopted by Peterson could be perceived by surveyed journalism undergraduate students as a decision-aid, while the *table only* approach we adopted was not perceived by surveyed financial analysts as a decision-aid.

An interesting difference between the results presented by Kelly [7] and our research is the fact that Kelly found no effect of display format on accuracy, while we found that graph (both column and line) enhanced accuracy in comparison with textual disclosure. Such inconsistencies might be explained either by differences in the complexity of tasks or by sampling issues. Kelly does not present the tasks performed by respondents (only describes it briefly), hence, we cannot compare task complexity (our tasks are depicted in S1 Text, S1 Table and S1 and S2 Figs). Kelly’s sample was comprised of only 18 undergraduate journalism students, and he does not describe if respondents received any financial incentive (our sample is comprised by 295 financial analysts, and we did not offer them any financial incentive).

To test H3, a chi-squared test was performed on males and females who were allocated to the overconfidence subsample. The results presented in Table 4 show that there is no difference in overconfidence between genders (*χ*^2^(1) < .001, *p* = .99).

**Table 4:** Number of correct answers by gender (Results from H3).

	Overconfidence		
Gender	No	Yes	Total
**Female**	65	36	101
** **	64.36%	35.64%	100%
**Male**	125	69	194
** **	64.43%	35.57%	100%
**Total**	190	105	295
	64.41%	35.59%	100%
Pearson *χ*^2^(1) < .001, p = .99			

Table 4 depicts the results from H3.

In order to test if the randomization procedure (biased coin) influenced the results of this experiment, we randomly selected a subsample of subjects who received the line graph condition with each “line graph” observation having a probability of .50 of being selected to this subsample, thus balancing the experiment. The results were robust, and significance levels and effects remained similar, as depicted in S1 Table. In addition, the results for H3 remained similar (*χ*^2^(1) = .286, *p* = .593). Thus, the unbalanced randomization did not significantly affect our results.

Table 5 summarizes the evidence collected in this study from all tested hypotheses.

**Table 5:** Summary of evidences.

Hypotheses	Supported?	p-value
H1A: Numerical information disclosed on tables enhances accuracy when compared to text (narrative) only disclosure.	No	.20
H1B: Numerical information disclosed on graphs enhances accuracy when compared to text (narrative) only disclosure.	Yes	.013 (column graph), .045 (line graph)
H2: Overconfident financial analysts commit more errors than non-overconfident ones.	Yes	< .01
H3: Male financial analysts are more overconfident than females.	No	.99

Table 5 summarizes results for all tested hypotheses.

## Discussion and Concluding Remarks

This study sheds some light on two points in the investigation of decision making of financial analysts: external and internal factors and how they interact with each other.

This research provides evidence that different ways of disclosing numerical information about flow could help or harm accuracy on decision making of accountants, financial analysts and investors [5, 8, 20, 25]. Accuracy provided by table disclosure was not different from that provided by textual disclosure, and it can be explained by the fact that both display *symbolic* information (numbers). Although Peterson [6] identified that tables enhance accuracy, she compared *text only* with *text plus table*; while we compared *table only* with *text only*. Column graph (p < .05) and line graph (p < .05) had significant positive impact on accuracy of financial analysts, enhancing their ability to correctly answer the questions. This may be attributed to the *spatial* information disclosure provided by those graphs. Due to the fact that time is a continuous variable, it is no surprise that the column and line graphs presented the best results compared to textual disclosure, because it may be easier to visualize the change in a continuous variable in a spatial disclosure rather than with a symbolic disclosure. The reason for this is that spatial disclosure provides people with clear baselines, and it is easier for them to find the maximum and the minimum. However, in tables the respondents must convert symbolic information to a spatial format in order to find both maximum and minimum [33]. Therefore, this study shows that for *continuous* variables to change, graphs that enhance users’ ability of perception and accuracy are the ones that communicate more *spatial* information (such as column and line graphs), which is consistent with Vessey’s theory of graphical disclosure [5].

The internal factor considered in this study is overconfidence, more specifically overplacement. Overconfidence was shown to harm accuracy on the interpretation of numerical information (across all types of information disclosure). This is consistent with previous research [8, 11, 26] showing that overconfidence harms the decision-making process.

Contrary to previous research [26–28], gender did not influence the overconfidence of individuals in our sample, suggesting that male and female financial analysts are equally overconfident when analyzing graphical representation of information. This may be an effect of self-selection. Maybe only overconfident females choose to be financial analysts, or perhaps this profession changes their overconfidence, making both genders homogeneous in this trait, as suggested by Barcellos, Cardoso and Aquino [9] and Hastie and Dawes [34].

Also, the type of information disclosure did not influence overconfidence, showing evidence that different types of disclosure with similar difficulty do not impact the overconfidence of financial analysts.

Most experimental research has internal validity but lacks external validity [30]. We aimed to achieve a higher degree of external validity surveying professional participants instead of students. Indeed, we intentionally selected a hypothetical non-accounting-oriented task (flow of people going in and out of a store during a 12 minute time period) in order to avoid experience bias, as previous institutionalized practices by the respondent could drive their response [31, 32].

There are multiple further implications of these results for practitioners and researchers. Due to the fact that overconfidence and graphical disclosure of numerical information influence financial analysts’ accuracy on decision making, training preparers of financial reports to identify which type of numerical disclosure best presents what they purport to represent and training auditors to audit graphs could enhance the quality (faithful representation) of financial reports. Furthermore, training financial analysts on graphical impression management and to assess their personal traits (such as overconfidence, self-consistency and confirming-evidence seekers), could enhance the quality of graphical interpretation and decision making.

Another angle of this research is how other external factors influence perception and decision making involving numerical information. One personal trait that was analyzed is overconfidence, but other “traps” may also exist, such as self-consistency (persisting on a wrong decision only because one cannot admit that his/her decision was wrong) and confirming-evidence seekers (one only seeks evidence that confirms his/her perception and beliefs) [13, 32]. Both “traps” can harm perception and decision making of financial analysts regarding graphical information.

There is evidence that people change their behavior when choosing for others versus when choosing for themselves [35]. Such evidence could also be moderated by the analysts’ level of overconfidence.

A fourth line of research is whether avoiding risks changes the perception of financial analysts regarding graphical information. One graph that shows a lot of ups and downs (big variance) could be regarded as worse than a graph showing consistent results (small variance), even if the expected return of the first is bigger than the second. Additionally, companies are increasing the use of videos and webcasts to present their reports. Hence, the impact of disclosure medium (e.g., printed versus video) on graphical perception and interpretation has not been investigated so far.

In summary, the field of study on how internal factors (personal traits) affect individuals’ perception on graphical information is growing in the area of behavioral accounting, and there are several research paths to be studied and questions to be answered in this area.

## Disclosure and Acknowledgments

While working as independent consultants for the BAA, two of the authors of this study (RLC and ACBA) developed the questionnaire which was applied to the respondents. Some data about accountants’ demographic characteristics were published under the title *Professional Accountants’ Profile*, ed. 2012/13 (CFC 2013). Besides the questions of interest to the BAA, we inserted some that were used exclusively in connection to our academic research. BAA agreed to allow us to have exclusive access to data related to these questions.

During the research the authors received funds from the National Research Council of Brazil (CNPq), Higher Personnel Advancement Coordination (CAPES), Rio de Janeiro State Research Foundation (FAPERJ) and from the Getulio Vargas Foundation (FGV). The abovementioned institutions do not have any responsibility of data collection and estimation. The views expressed in this article represent the authors’ point of view, and not from any institution.

We would like to thank Leonardo P. Barcellos for his help, insightful discussions and literature suggestions, and Luiz Claudio F. S. Junior for his help with statistical methodology. The analyses in this paper were performed using the Stata® software version 13. Database (S1 File) and do-file (S2 and S3 Files) together with the statistical tests and results are available for replication in S2 Text.

A previous version of this paper was presented in the 2015 AdCont Conference and 2016 USP Conference, but the authors made significant changes in data analyses, references and hypotheses before submitting it to the this Journal. Concerning those conferences, we also thank José Alonso Borba, Adolfo Coutinho, Adriano Rodrigues and Marcelo Álvaro for their suggestions. We also thank the two anonymous reviewers for their insightful suggestions and corrections.

## Supporting Information

S1 Fig Line graph experimental condition. S1 Fig depicts the line graph experimental condition manipulated between-subjects.
(TIF)

S2 Fig Column graph experimental condition. S2 Fig depicts the column graph experimental condition manipulated between-subjects.
(TIF)

S1 File STATA database. S1 File contains the database.
(DTA)

S2 File STATA do-file for main results. S2 File contains the commands for main analysis of database.
(DO) Click here for additional data file.

S3 File STATA do-file for robustness check. S3 File contains the commands for robustness check.
(DO) Click here for additional data file.

S1 Table Table experimental condition. S1 Table depicts the table experimental condition manipulated between-subjects.
(DOCX)

S2 Table Robustness check for balanced subsamples. S2 Table depicts the robustness check results (balanced sample) from the ordered logit model, ordered probit and OLS regression. The balance sample consists of randomly selected subsample of line graph respondents and all respondents of the other three conditions.
(DOCX)

S1 Text Text experimental condition. S1 Text depicts the textual (narrative) experimental condition manipulated between-subjects.
(DOCX)

S2 Text Replication instructions for “A Graph is Worth a Thousand Words”. S2 Text describes the database and presents a replication instructions for the results featured in the paper.
(PDF)
