# Supplementary figures and images for: A Graph is Worth a Thousand Words: How Overconfidence and Graphical Disclosure of Numerical Information Influence Financial Analysts Accuracy on Decision Making
Source: PLoS One. 2016 Aug 10;11(8):e0160443. doi: 10.1371/journal.pone.0160443 (PMC4980045; doi:10.1371/journal.pone.0160443)

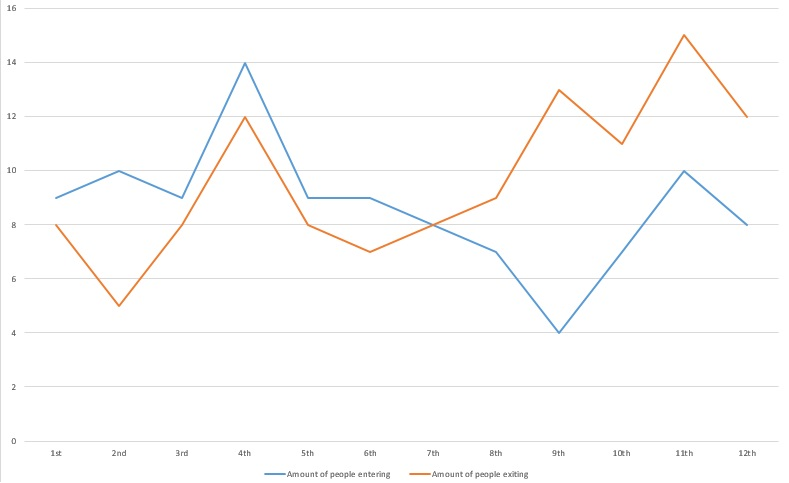

Supplement: S1 Fig — depicts the line graph experimental condition manipulated between-subjects. (TIF) [file pone.0160443.s001.tif]

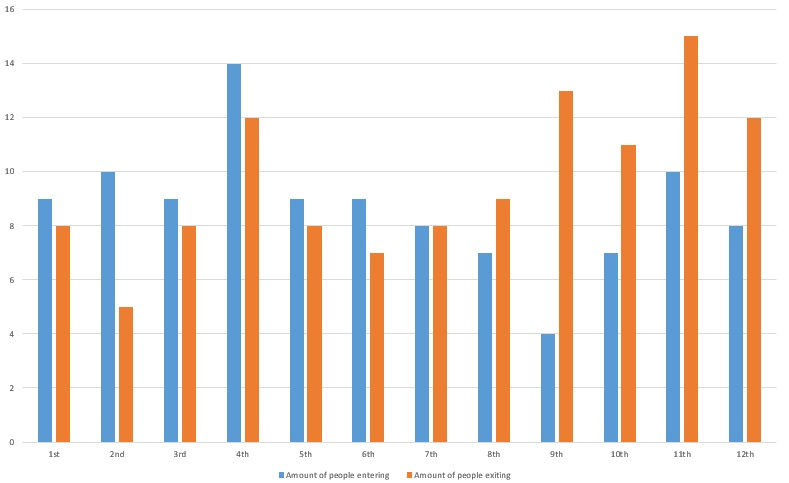

Supplement: S2 Fig — depicts the column graph experimental condition manipulated between-subjects. (TIF) [file pone.0160443.s002.tif]
