# Supplementary material for: A Graph is Worth a Thousand Words: How Overconfidence and Graphical Disclosure of Numerical Information Influence Financial Analysts Accuracy on Decision Making
Source: PLoS One. 2016 Aug 10;11(8):e0160443. doi: 10.1371/journal.pone.0160443 (PMC4980045; doi:10.1371/journal.pone.0160443)
Supplement: S1 Table — depicts the table experimental condition manipulated between-subjects. (DOCX) [file pone.0160443.s006.docx]

**S1 Table: Table experimental condition**

| Minute | Amount of people entering | Amount of people exiting |
| --- | --- | --- |
| 1^st^ | 9 | 8 |
| 2^nd^ | 10 | 5 |
| 3^rd^ | 9 | 8 |
| 4^th^ | 14 | 12 |
| 5^th^ | 9 | 8 |
| 6^th^ | 9 | 7 |
| 7^th^ | 8 | 8 |
| 8^th^ | 7 | 9 |
| 9^th^ | 4 | 13 |
| 10^th^ | 7 | 11 |
| 11^th^ | 10 | 15 |
| 12^th^ | 8 | 12 |
| S1 Table depicts the table experimental condition manipulated between-subjects. | | |
