# Supplementary material for: A Graph is Worth a Thousand Words: How Overconfidence and Graphical Disclosure of Numerical Information Influence Financial Analysts Accuracy on Decision Making
Source: PLoS One. 2016 Aug 10;11(8):e0160443. doi: 10.1371/journal.pone.0160443 (PMC4980045; doi:10.1371/journal.pone.0160443)
Supplement: S2 Table — depicts the robustness check results (balanced sample) from the ordered logit model, ordered probit and OLS regression. The balance sample consists of randomly selected subsample of line graph respondents and all respondents of the other three conditions. (DOCX) [file pone.0160443.s007.docx]

**S2 Table: Robustness check for balanced subsamples**

|  | **Ordered Logit** | | | **Ordered Probit** | | | **OLS** | | |
| --- | --- | --- | --- | --- | --- | --- | --- | --- | --- |
|  | **Model 1** | **Model 2** | **Model 3** | **Model 4** | **Model 5** | **Model 6** | **Model 7** | **Model 8** | **Model 9** |
| Overconfidence | -.750** | -.697** | -.724** | -.464** | -.432** | -.444** | -.270*** | -.250** | -.254** |
|  | (.311) | (.316) | (.318) | (.181) | (.183) | (.184) | (.097) | (.097) | (.097) |
| Line Graph |  | .901** | .907** |  | .511** | .520** |  | .272** | .270** |
|  |  | (.436) | (.436) |  | (.254) | (.255) |  | (.134) | (.134) |
| Column Graph |  | 1.048** | 1.032** |  | .532** | .514** |  | .256** | .249* |
|  |  | (.422) | (.428) |  | (.240) | (.241) |  | (.127) | (.127) |
| Table |  | .546 | .526 |  | .324 | .308 |  | .199 | .190 |
|  |  | (.424) | (.425) |  | (.253) | (.254) |  | (.139) | (.139) |
| Gender (1 = male) |  |  | -.326 |  |  | -.187 |  |  | -.083 |
|  |  |  | (.344) |  |  | (.197) |  |  | (.098) |
| N | 226 | 226 | 226 | 226 | 226 | 226 | 226 | 226 | 226 |
| Chi² / F † | 5.72*** | 13.17*** | 14.09** | 6.50*** | 12.58** | 13.49** | 7.70*** | 3.26** | 2.47** |
| Pseudo-R**²** / AdjR² ‡ | 0.017 | 0.039 | 0.042 | 0.019 | 0.038 | 0.040 | 0.033 | 0.039 | 0.037 |
| Standard Errors in parenthesis. | | | | | | | | | |
| * p<.1, ** p<.05, *** p<.01 | | |  |  |  |  |  |  |  |
| All interactions in all models are nonsignificant.  † Chi²-test for the Ordered Logit and Ordered Probit models and F-test for the OLS models.  ‡ Pseudo-R² for the Ordered Logit and Ordered Probit models and AdjR² for the OLS models. | | | | | | | | | |

S2 Table depicts the robustness check results (balanced sample) from the ordered logit model, ordered probit and OLS regression. The balance sample consists of randomly selected subsample of line graph respondents and all respondents of the other three conditions.
