# Supplementary material for: A Graph is Worth a Thousand Words: How Overconfidence and Graphical Disclosure of Numerical Information Influence Financial Analysts Accuracy on Decision Making
Source: PLoS One. 2016 Aug 10;11(8):e0160443. doi: 10.1371/journal.pone.0160443 (PMC4980045; doi:10.1371/journal.pone.0160443)
Supplement: S1 Text — depicts the textual (narrative) experimental condition manipulated between-subjects. (DOCX) [file pone.0160443.s008.docx]

**S1 Text: Text experimental condition**

In the first minute, 9 people entered and 8 exited from the store. In the second minute, 10 people entered and 5 exited from the store. In the third minute, 9 people entered and 8 exited from the store. In the fourth minute, 14 people entered and 12 exited from the store. In the fifth minute, 9 people entered and 8 exited from the store. In the sixth minute, 9 people entered and 8 exited from the store. In the seventh minute, 8 people entered and 8 exited from the store. In the eighth minute, 7 people entered and 9 exited from the store. In the eighth minute, 7 people entered and 9 exited from the store. In the ninth minute, 4 people entered and 13 exited from the store. In the tenth minute, 7 people entered and 11 exited from the store. In the eleventh minute, 10 people entered and 15 exited from the store. In the twelfth minute, 8 people entered and 12 exited from the store.

S1 Text depicts the textual (narrative) experimental condition manipulated between-subjects.
