# Supplementary material for: A Graph is Worth a Thousand Words: How Overconfidence and Graphical Disclosure of Numerical Information Influence Financial Analysts Accuracy on Decision Making
Source: PLoS One. 2016 Aug 10;11(8):e0160443. doi: 10.1371/journal.pone.0160443 (PMC4980045; doi:10.1371/journal.pone.0160443)
Supplement: S2 Text — describes the database and presents a replication instructions for the results featured in the paper. (PDF) [file pone.0160443.s009.pdf]

Replication Instructions for "A Graph is Worth a Thousand Words"

January 30, 2016

Abstract

This is an Appendix to the "A Graph is Worth a Thousand Words" article, detailing the database and the replication instructions for the results featured in the paper.

In addition to this document, two other files are needed for the replication:

- `experiment-graphtype.dta`, which contains the experimental data, and
- `do-file-for-replication.do`, which contains the statistical code.

The `.dta` file has a total of eight variables, as follow:

- `id`, participant's ID (1-295 range);
- `gender`, dummy (0 = male, 1 = female);
- `age`, participant's age in years;
- `residence`, participant's city of residence dummy (0 = State Capital, 1 = otherwise);
- `graphtype`, this is the experimental assignment with four conditions (line graph = 1, column graph = 2, table = 3, text = 4);
- `overconfidence`, dummy for overconfidence (0 = yes, 1 = no);

- `q1`, dummy for the first answer (0 = wrong, 1 = right);
- `q2`, dummy for the second answer (0 = wrong, 1 = right).

Both files are in Stata® 13 versions. For usage in previous Stata® versions the `use13` command is necessary for the `.dta` file, while the `.do` file works normally in any version.
